# Case Report: Recombinant human growth hormone therapy in a patient with spondyloepiphyseal dysplasia, Kondo-Fu type

**DOI:** 10.3389/fped.2023.1068718

**Published:** 2023-02-03

**Authors:** Congli Chen, Jin Wu, Ying Liu

**Affiliations:** ^1^ Department of Pediatrics, West China Second Hospital, Sichuan University, Chengdu, China; ^2^ Key Laboratory of Birth Defects and Related Diseases of Women and Children (Sichuan University), Ministry of Education, Chengdu, China

**Keywords:** spondyloepiphyseal dysplasia, Kondo-Fu type, MBTPS1, genotype, phenotype, rhGH therapy

## Abstract

**Background:**

Variants in membrane-bound transcription factor peptidase, site 1 (MBTPS1) gene, can result in clinically rare spondyloepiphyseal dysplasia of Kondo-fu type (OMIM #618392, SEDKF), Silver–Russell syndrome, and CAOP (cataract, alopecia, oral mucosal disorder, and psoriasis-like) syndrome.

**Case presentation:**

A 6-year-old Chinese male child diagnosed with SEDKF underwent 3 years of growth hormone therapy. A genetic examination revealed two new nonsense variants in the MBTPS1 gene on chromosome 16q23-q24 with compound heterozygotes c.1589(exon12)A > G and c.163(exon2)G > A.

**Conclusion:**

The MBTPS1 gene c.1589(exon12)A > G and c.163(exon2)G > A on chromosome 16q23-q24 is associated with SEDKF. Growth hormone therapy can repair growth retardation in patients with spondyloepiphyseal dysplasia, Kondo-Fu type; however, more evidence of such patient cases is required to support this hypothesis.

## Background

Site-1 protease (S1P) encoded by the MBTPS1 (membrane-bound transcription factor peptidase, site 1) gene regulates cholesterol and lipid homeostasis *via* cleavage of substrates at non-basic residues, endoplasmic reticulum function, and lysosome biogenesis in mice and cultivated cells (1–4). MBTPS1 is also essential for the transcription of bone matrix and mineralization, expressing proteins including type XI collagen, Phex, Dmp1, fibronectin, and fibrillin in bone osteoblasts and osteocytes (5).

Variants in MBTPS1 may be associated with lysosomal dysfunction (6, 7), reportedly resulting in a variety of phenotypes. Spondyloepiphyseal dysplasia, Kondo-fu type (OMIM #618392, SEDKF), was reported in 2018 (6) and 2020 (8) and was characterized by severely retarded growth, skeletal anomalies, dysmorphic features, epilepsy, craniosynostosis, and elevated levels of blood lysosomal enzymes. In 2020, Meyer et al. (9) reported a patient with variants in MBTPS1 presenting Silver–Russell syndrome, characterized by a heterogeneous congenital growth retardation syndrome. In 2022, Chen et al. (10) illustrated two unrelated patients with CAOP (cataract, alopecia, oral mucosal disorder, and psoriasis-like) syndrome caused by variants in MBTPS1. In addition, hyperCKemia and focal myoedema were found to be related to variants in MBTPS1. The number of human diseases with site-1 protease deficiency is extremely rare, and totally, only two cases of SEDKF, one case of Silver–Russell syndrome, two cases of CAOP syndrome, and one patient presenting with hyperCKemia and focal myoedema have been reported (6, 8–11). In addition, the precise effect of recombinant human growth hormone (rhGH) therapy on the height of patients with growth retardation with MBTPS1 variants remains unclear.

Herein, we report a case of a 6-year-old Chinese boy with an analogous SEDKF phenotype, including skeletal dysplasia, distinctive facies, and decreased body weight with a novel homozygous nonsense variant in MBTPS1, and who received rhGH therapy for 3 years. To the best of our knowledge, he is the third individual with SEDKF. Therefore, the existence of this rarely diagnosed disease with variants in MBTPS1 and the exact influence of rhGH therapy on the height of patients need to be elucidated further.

## Case presentation

The patient was born as the first child of unrelated and healthy parents. His mother was 28 years old and the father was 34 years old when the child was delivered. He was a term baby through spontaneous vaginal delivery in the year 2016 after an uneventful pregnancy, except for a successful rescue of meconium aspiration syndrome, and had no family history of the disease. He received artificial feeding after birth with 600–700 ml of milk per day, and supplementary food was added at the age of 5 months. At 4 months of age, he presented with motor retardation with poor head-up ability and low muscular tension but no other abnormal signs of hearing, expressive language, and intelligence development.

At the age of 11 months, he presented with poor appetite and slow growth in height and weight but had no manifestation of emesis, diarrhea, or jaundice. A food intolerance test revealed that he was positive (++) for milk protein. At the age of 35 months, he underwent surgical lens removal for a congenital lamellar cataract.

**Figure 1:**
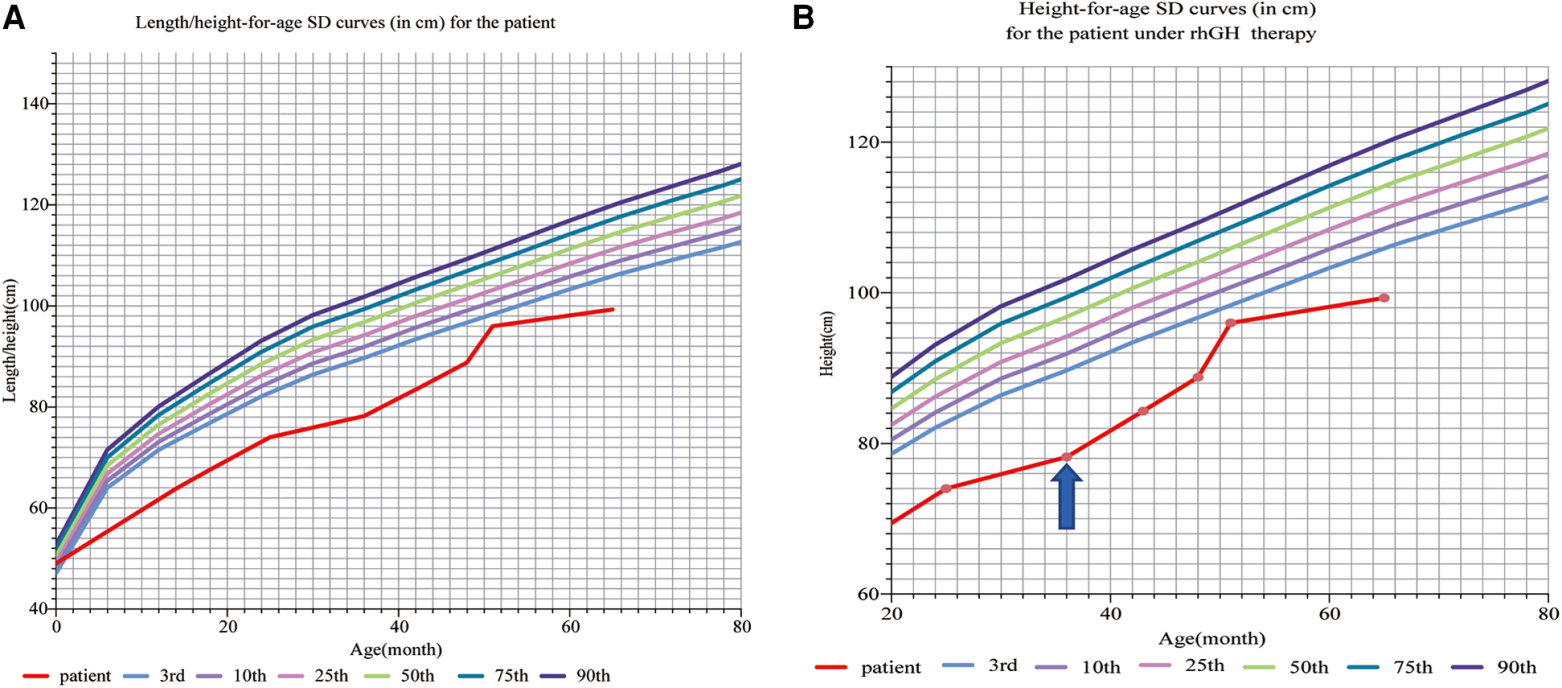
(**A**) Length/height-for-age SD curves (in cm) for a patient before rhGH therapy: according to the Chinese children’s growth standards, the proband’s born weight (BW) is 2,680 g (−1.59SD, P5.5), and birth length is 49 cm (0.78SD, P21.7). At the age of 14 months, the patient presented with a short stature and a slightly sloping shank, his height was 63.80 cm (−5.3SD, ≤P0.1), and his weight was 6.00 kg (−3.8SD, ≤P0.1). At the age of 36 months, his height was 78.2 cm (−4.84SD, ≤P0.1), weight was 9 kg (−3.33SD, ≤P0.1), and BMI was 14.72 (−0.62SD, P26.7). (**B**) Height-for-age SD curves (in cm) for the patient under rhGH therapy. The patient received rhGH therapy at a dosage of 0.15–0.2 IU/kg/d ih at the age of 36 months (the arrow points to). At the age of 36 months, his height was 78.2 cm (−4.84SD, ≤P0.1), weight was 9 kg (−3.33SD, ≤P0.1), and BMI was 14.72 (−0.62SD, P26.7). After 3 years of rhGH therapy, the patient showed rapid growth, following which his height was 99 cm (−3.96SD, ≤P0.1) and weight was 12.80 kg (−2.84SD, ≤P0.1) at the age of 65 months. rhGH, recombinant human growth hormone.

The proband’s born weight (BW) was 2,680 g (−1.59SD, P5.5), and birth length was 49 cm (−0.78SD, P21.7). At the age of 14 months, he presented with a short stature and a slightly sloping shank; his height was 63.80 cm (−5.3SD, ≤P0.1), and his weight was 6.00 kg (−3.8SD, ≤P0.1). At the age of 35 months, his height was 78.2 cm (−4.86SD, ≤P0.1), weight was 9 kg (−3.33SD, ≤P0.1), and BMI was 14.72 (−0.62SD, P26.7). The growth standards of Chinese children were developed on the basis of a study of their physical development in nine provinces and cities in the year 2005 and published in the 7th issue of the *Chinese Journal of Pediatrics* in 2009, and since then, our physical measurements have been assessed using these standards (Figure 1A).

**Figure 2:**
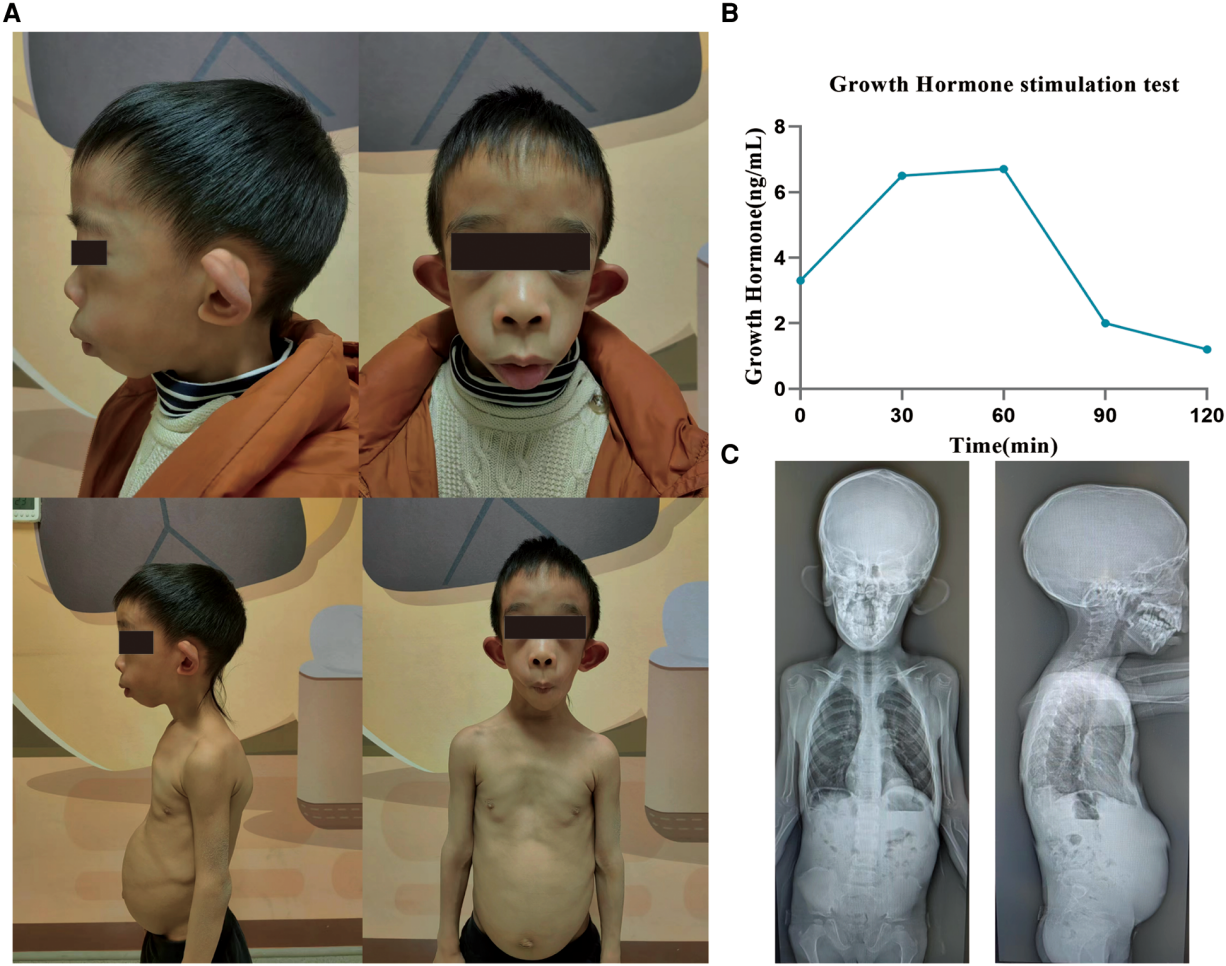
Clinical characteristics of a patient with the variant in MBTPS1. (**A**) The patient shows distinctive facies with macrotia, retromicrognathia, sternal malformation, protruding abdomen, and kyphoscoliosis. (**B**) The growth hormone stimulation test shows that under the stimulation of arginine and levodopa, the growth hormone level peaks at 60 min: 6.7 ng/ml (peak >10 indicates normal levels, 5–10 indicates part deficiency, and <5 indicates deficiency). (**C**) Radiographs at the age of 6 years. A notable straightened physiological curvature of the cervical, thoracic, and lumbar spine, irregular morphology of cones, reduced bone density of the cones, C2–C3 and C3–C4 intervertebral disc space narrowing, and bilateral shallow acetabulum. MBTPS1, membrane-bound transcription factor peptidase, site 1.

The boy showed distinctive facies with macrotia, retromicrognathia, sternal malformation, protruding abdomen, and kyphoscoliosis (Figure 2A).

A growth hormone stimulation test showed that under the stimulation of arginine and levodopa, the growth hormone level peaked at 60 min: 6.7 ng/ml (Figure 2B). Alkaline phosphatase was slightly above the reference limit (321 U/L, 161.9–296.4). No abnormality was detected in the liver and kidney function, electrolytes, blood gas analysis, parathyroid hormone, and thyroid function tests; also, IGF-1 levels were normal.

Radiographs taken at the age of 6 years revealed a straightened physiological curvature of the cervical, thoracic and lumbar spine, irregular morphology of cones, reduced bone density of the cones, C2–C3 and C3–C4 intervertebral disc space narrowing, and bilateral shallow acetabulum (Figure 2C).

Brain magnetic resonance imaging (MRI), echocardiography, and renal ultrasonography were unremarkable.

**Figure 3:**
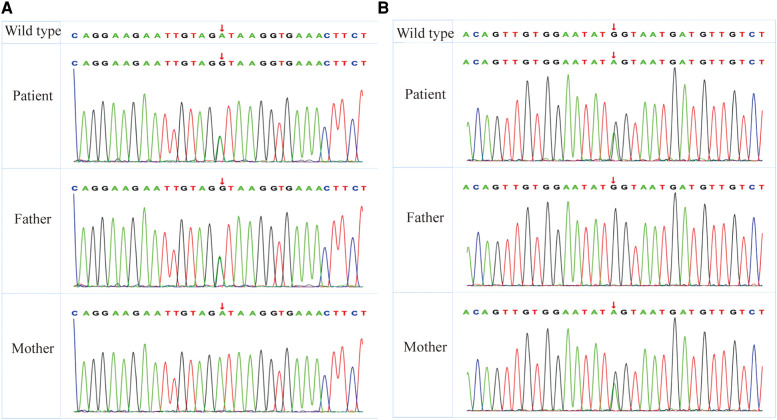
WES reveals heterozygous MBTPS1 p.Asp530Gly (exon12; c.1589A > G) and p.Glu55Lys (exon2; c.163T > C) variants in the patient. (**A**) The heterozygous variant of p.Asp530Gly (exon12; c.1589A > G) was found in his father, whereas his mother had the wild type. (**B**) The other heterozygous variant of p.Glu55Lys (exon2; c.163T > C) was found in his mother, whereas his father had the wild type. WES, whole exome sequencing; MBTPS1, membrane-bound transcription factor peptidase, site 1.

A chromosome examination showed that the karyotype was 46, XY, containing benign copy number variations. By whole exome sequencing (WES), c.1589(exon12)A > G in chr16:84108206 and c.163(exon2)G > A in chr16:84135226 were detected in the patient. The heterozygous variant of c.1589(exon12)A > G was found in his father, whereas his mother had the wild type (Figure 3A). The other heterozygous variant c.163(exon2)G > A was found in his mother, whereas his father had the wild type (Figure 3B). Both variants’ minor allele frequencies were <0.05% and were not listed in the ClinVar (https://clinicalgenome.org) and Genome AD databases. Bioinformatics protein function prediction software SIFT, PolyPhen_HDIV, PolyPhen2_HVAR, PROVEAN, MutationTaster, and GERP were used to predict the protein function. The result of c.1589(exon12)A > G was pathogenic moderate (PM2), and the results of c.163(exon2)G > A were PM2 and pathogenic supporting (PP3). According to ACMG guidelines (12), both c.1589(exon12)A > G and c.163(exon2)G > A were classified to be of uncertain significance, respectively.

With the analogous SEDKF phenotype, including skeletal dysplasia, distinctive facies, decreased body weight, and growth retardation and WES genetic test revealing c.1589(exon12)A > G and c.163(exon2)G > A variant in MBTPS1, the proband was diagnosed with SEDKF.

The patient received rhGH therapy at a dosage of 0.15–0.2 IU/kg/d ih at the age of 3 years, in addition to basic therapies administering calcium, vitamin A, and vitamin D.

After 3 years of rhGH therapy, the patient showed rapid growth, following which his height was 99 cm (−3.96SD, ≤P0.1) and weight 12.80 kg (−2.84SD, ≤P0.1) (Figure 1B), and his upper segment was 54 cm. Strikingly, the IGF-1 levels and thyroid function were normal.

## Discussion and conclusion

MBTPS1 encodes a member of the subtilisin-like proprotein convertase family (2), which includes proteases that process protein and peptide precursors trafficking through regulated or constitutive branches of the secretory pathway (7). S1P (encoded by MBTPS1) deficiency causes a partial impairment of mannose-6-phosphate-dependent Golgi-to-lysosome transport of lysosomal enzymes and may be associated with lysosomal dysfunction (7).

Two patients with SEDKF, one patient with Silver–Russell syndrome, two patients with CAOP syndrome, and one patient with hyperCKemia and focal myoedema presented with variants in MBTPS1. Patients with SEDKF showed severe retarded growth, skeletal anomalies, dysmorphic features, epilepsy, craniosynostosis, and elevated levels of blood lysosomal enzymes (6, 8). Kondo et al. discovered shortened tubular bones and delayed ossification of epiphyses and carpal bones, while Carvalho et al. uncovered diffuse osteoporosis, ovoid lumbar vertebrae bodies, irregular cervical vertebrae bodies, long bones with irregular metaphysis and epiphyses, mildly enlarged metaphysis, small tubular epiphyses, and copper beaten skulls. The patient with Silver–Russell syndrome presented with a heterogeneous congenital growth retardation, including a triangular face, large ears, funnel chest, kyphosis, pes valgus, sandal grooves, cataract, unilateral inguinal hernia, and motor milestone delay (9). The two unrelated patients presented with CAOP syndrome (10).

In addition, the proband had something in common with the other patients with variants in MBTPS1. He was featured as small for gestational age, short stature with growth drop-off at 14 months, poor bodyweight gain, characteristic facies such as a prominent forehead and posteriorly rotated ears, delayed ossification of carpal bones, and cataract and surgical lens removal for congenital lamellar cataract (Table 1).

**Table 1:** Characteristics of patients with MBTPS1 gene variants in the literature.

Reference	Kondo et al. (2018)	Schweitzer et al. (2019)	Meyer et al. (2020)	Carvalho et al. (2020)	Chen et al. (2022)	Chen et al. (2022)	Present study
Patient number	1	2	3	4	5	6	7
Skeletal dysplasia	+	−	+	+	−	−	+
Sternal malformation	+	NA	+	+	−	−	+
Short stature	+	NA	+	+	−	−	+
Kyphoscoliosis	+	NA	NA	−	−	−	+
Retromicrognathia	+	NA	+	+	−	−	+
Large ears	+	NA	+	+	−	−	+
Cataract	NA	NA	+	+	+	+	+
Protruding abdomen	NA	NA	NA	+	−	−	+
Elevated blood lysosomal enzymes	+	NA	+	+	−	ND	ND
Elevated hepatic transaminase	−	+	−	+	NA	NA	−
HyperCKemia	−	+	NA	−	−	ND	−
Focal myoedema	NA	+	NA	NA	−	ND	−
Cutaneous lesions	−	NA	NA	NA	+	+	−
Mucosal lesions	−	NA	NA	NA	+	ND	−
Associated variation	Exon 3; c.285dupT/exon 9; c.1094A > G	Exon 23; c.3007C > T	c.1094A > G homozygosity	Exon 22; c.2948G > A	c.1064T > G/c.3157T > C	c.3159A > T/c.2072-2A > T	Exon12; c. 1589A > G/exon2; c.163G > A

MBTPS1, membrane-bound transcription factor peptidase, site 1; ND, not detected; NA, not available.

Being the first reported case of SEDKF in China, this patient was found to harbor a compound heterozygous variant in the MBTPS1 gene (OMIM #603355) on chromosome 16q23-q24 through genetic testing. Because the child was young, there was no obvious spinal change. Also, food intolerance with diarrhea after ingestion of milk was noted. Unlike other children, there was no apparent carpal retardation in this child.

The patient with SEDKF, reported by Kondo et al. in 2018, received growth hormone replacement therapy for 1 year but discontinued it as a result of limited response. Silver–Russell syndrome can also be treated with growth hormone therapy, but it is not easy to achieve normal height after treatment. Due to the proband’s short stature and growth retardation, a growth hormone stimulation test was conducted on this patient, and the results suggested a dysfunction of growth hormone secretion, but the child’s IGF-1 level was normal. Although he received rhGH therapy for 3 years, its efficacy might be limited due to an underlying genetic defect in the IGF-1 function. However, after treatment, the child’s height increased from −4.86SD to −3.96SD, indicating that the growth hormone had specific benefits in increasing the height of such patients.

However, due to a lack of experimental examinations, a comparison of the increasing levels of lysosomal enzyme with other cases is not possible.

Here, we reported the case of a patient with variants in the MBTPS1 gene, c.1589(exon12)A > G and c.163(exon2)G > A, associated with SEDKF. As the patient showed a relatively significant height increase with growth hormone treatment, it can be assumed that growth hormone therapy can repair growth retardation in patients with spondyloepiphyseal dysplasia, Kondo-Fu type; however, more evidence of such patient cases is required to support this hypothesis.

## Data Availability

The original contributions presented in the study are included in the article/Supplementary Material, further inquiries can be directed to the corresponding authors.
